# Neural Reduction of Image Data in Order to Determine the Quality of Malting Barley

**DOI:** 10.3390/s21175696

**Published:** 2021-08-24

**Authors:** Piotr Boniecki, Barbara Raba, Agnieszka A. Pilarska, Agnieszka Sujak, Maciej Zaborowicz, Krzysztof Pilarski, Dawid Wojcieszak

**Affiliations:** 1 Department of Biosystems Engineering, Poznań University of Life Sciences, ul. Wojska Polskiego 50, 60-627 Poznań, Poland; bonie@up.poznan.pl (P.B.); barbara.raba@claas.com (B.R.); agnieszka.sujak@up.poznan.pl (A.S.); maciej.zaborowicz@up.poznan.pl (M.Z.); pilarski@up.poznan.pl (K.P.); dawid.wojcieszak@up.poznan.pl (D.W.); 2 Department of Food Technology of Plant Origin, Poznań University of Life Sciences, ul. Wojska Polskiego 31, 60-624 Poznań, Poland

**Keywords:** computer analysis of the digital image, neural compression of graphical data, classification of malting barley quality

## Abstract

Image analysis using neural modeling is one of the most dynamically developing methods employing artificial intelligence. The feature that caused such widespread use of this technique is mostly the ability of automatic generalization of scientific knowledge as well as the possibility of parallel analysis of the empirical data. A properly conducted learning process of artificial neural network (ANN) allows the classification of new, unknown data, which helps to increase the efficiency of the generated models in practice. Neural image analysis is a method that allows extracting information carried in the form of digital images. The paper focuses on the determination of imperfections such as contaminations and damages in the malting barley grains on the basis of information encoded in the graphic form represented by the digital photographs of kernels. This choice was dictated by the current state of knowledge regarding the classification of contamination that uses undesirable features of kernels to exclude them from use in the malting industry. Currently, a qualitative assessment of kernels is carried by malthouse-certified employees acting as experts. Contaminants are separated from a sample of malting barley manually, and the percentages of previously defined groups of contaminations are calculated. The analysis of the problem indicates a lack of effective methods of identifying the quality of barley kernels, such as the use of information technology. There are new possibilities of using modern methods of artificial intelligence (such as neural image analysis) for the determination of impurities in malting barley. However, there is the problem of effective compression of graphic data to a form acceptable for ANN simulators. The aim of the work is to develop an effective procedure of graphical data compression supporting the qualitative assessment of malting barley with the use of modern information technologies. Image analysis can be implemented into dedicated software.

## 1. Introduction

Modern technologies used in the agri-food industry frequently help to improve the efficiency of production processes, which allow increasing the revenues of enterprises and thus strengthening their position on the market [1]. Currently, the challenge for this branch of the economy is the production of agri-food products characterized by the best parameters in terms of quality, while maintaining optimal costs of the production and distribution of the processed biological material [2,3,4]. It is therefore essential to search for new, increasingly sophisticated methods and technologies to meet these requirements. A new solution, which is still being developed, is the use of the so-called machine vision that can replace human work in both qualitative and quantitative assessment processes. The undoubted advantage of using this type of method is maintaining the objectivity of the assessment, increasing its speed and, importantly, eliminating the expert’s fatigue [5,6].

The work focuses on the determination of malting barley grain (Latin: *Hordeum vulgare*) impurities. This choice was dictated by unsatisfactory reports concerning the state of the art of classification methods currently used in the malting industry [7,8]. The qualitative evaluation of kernels is conducted by certified malt workers acting as experts. They manually separate the impurities from the malting barley sample and then calculate the percentage of (predefined) impurity groups. The analysis of the problem area shows that there is no effective method of qualitative identification of barley kernels, e.g., with the use of information technology [9,10]. Therefore, it is justified to investigate the possibility of using modern methods of artificial intelligence to determine the contamination or damages of malting barley [11,12,13].

The work aimed to develop a new, effective method of malting barley quality assessment with the use of neural image analysis techniques [14,15,16]. The biological material presented in the form of digital images was classified. In this context, the process of compression of graphical empirical data was analyzed, especially with the use of self-associative neural networks (SANN).

## 2. Materials and Methods

### 2.1. Materials

The object of the research was the malting barley used for the production of malt. In Poland, 32 registered malting barley varieties dominate, including 29 spring and 3 winter varieties (the small number of the latter is due to reduced resistance of barley to low temperatures) [7]. In order to supplement the range, malt houses often import other varieties of barley, e.g., from European Union countries. This is due to the precise requirements imposed on malt houses by international brewing concerns. In practice, this means combining malting barley varieties until the defined and expected brewing parameters are achieved. The material used in the research was obtained from the malt house of Soufflet Polska Limited Company (Poznań, Poland).

Experiments were conducted involving a popular spring variety of malting barley—Sebastian, characterized by good technological features [17]. The most important technological characteristics of malt barley are: extractivity, wort viscosity, and Kolbach index (see Table 1).

- Extractivity—it is expressed as the sum of extractable substances that are transmitted to the solution during the conventional (infusion) mashing process. Extractivity of malt depends on the quality of the used kernels and ranges from 76% to 82%. In dark malt, it is by approx. 4% lower. Based on extractivity, productivity is calculated, and the amount of malt used for brewing a particular amount of beer is determined;
- Wort viscosity—a parameter that characterizes the extent of malt modification. The standard for wort viscosity is from 1.51 to 1.63 mPa×s. Higher values reveal insufficient decomposition of cell walls, which may lead to difficulties during the filtration of wort and beer;
- Kolbach index—an indicator referring to the amount of protein extracted from malt in the process of laboratory wort preparation in congress mash. It indicates what percent of malt protein can be found in the wort as a result of mashing.

It is customary to determine the brewing value synthetically (column 1, Table 1) [18].

**Figure 1:**
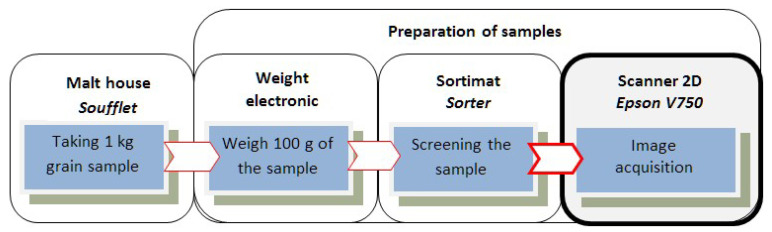
Scheme of sample preparation for barley kernel image acquisition.

The scheme of preparation of the samples of the kernel of the above-selected malting barley is shown in Figure 1.

**Figure 2:**
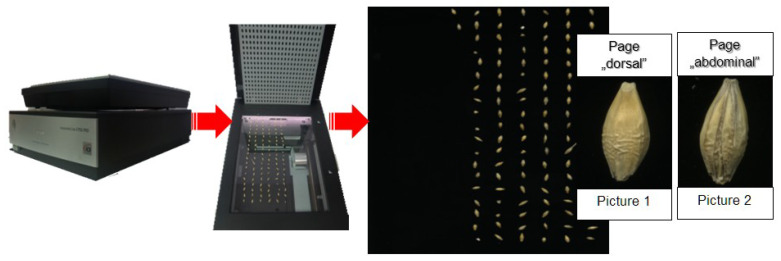
The process of acquiring digital images of kernels: “dorsal” and “ventral” side.

To obtain digital images representing seeds of the *Sebastian* variety, an EPSON V750-M Pro 2D flatbed scanner was used, which allowed for obtaining high-quality sets of photos. The applied parameters of the scanner were as follows: optical resolution—6400 dpi, optical density—4 Dmax, color depth—input/output 48 Bit Color, converter—CCD, and light source—cold cathode fluorescent lamp [17] (see Figure 2).

**Figure 3:**
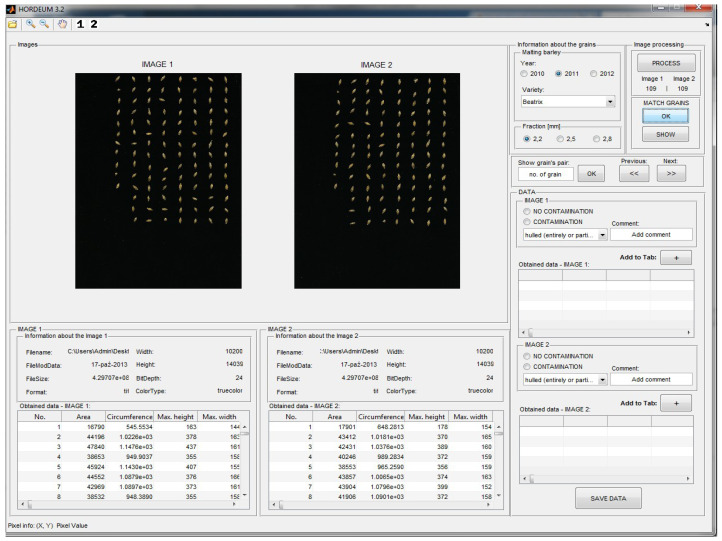
Screenshots of selected windows of the “*Hordeum* v. 3.2” IT system.

A total of 176 digital images of malting barley kernels of the cultivar Sebastian were acquired. For the processing and analysis of digital images of kernels, the original IT system “*Hordeum* v.3.2” (see Figure 3) was developed, which is not only dedicated to image processing and analysis but is also a useful tool for the generation of the teaching files for ANN. For the design and construction of the above-mentioned application, GUI (graphical user interface) was used as a standard implemented in the MATLAB 2014b environment (Figure 3). Elements of the Statistica v. 10 statistical package were used to create a neural compressor of graphic empirical data. The standard procedure implemented in the “STATISTICA Neural Networks PL—autoassociative networks—nonlinear dimension reduction” module by StatSoft, described in the Section 2.2 (pages: 7 and 8), was used.

The analyses distinguished 12 types of imperfection (Table 2) features. The analysis aimed to obtain a precise description of the impurity groups of barley kernels, which were then defined by appropriate characteristic parameters. The most common groups of imperfections occurring in a given batch of barley during the production process in malt houses were considered.

**Figure 4:**
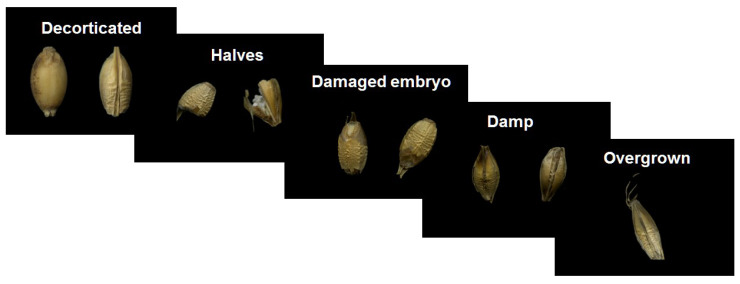
Exemplary images of imperfections of kernels or types of contamination.

Figure 4 presents examples of 5 typical imperfections of malting barley kernels.

**Figure 5:**
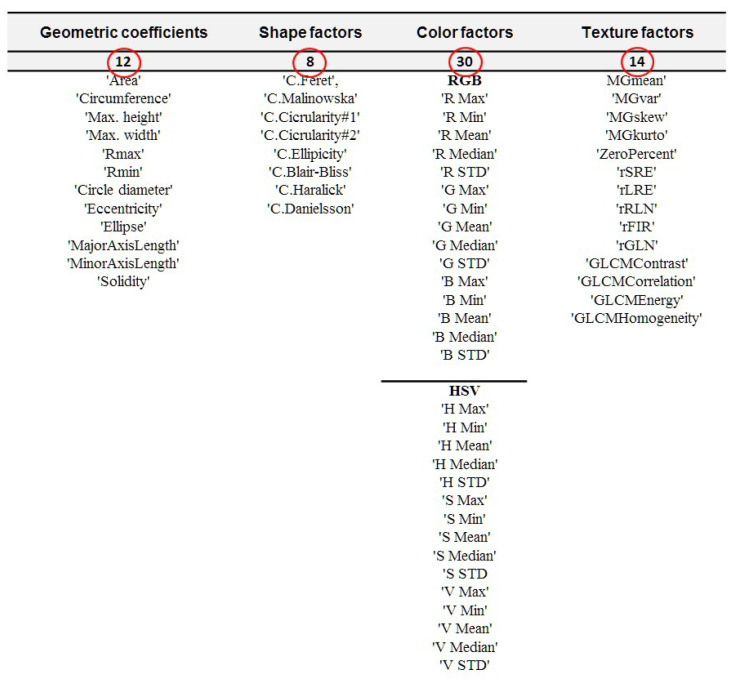
Specification of the barley varieties.

The created IT system “*Hordeum* v.3.2” (Figure 3) was equipped with a module designed to generate graphic parameters characterizing digital images. Sixty-four standard descriptors representing kernel graphics were selected, which are further input variables of training files necessary in the process of creation of ANN models (see Figure 5).

**Figure 6:**
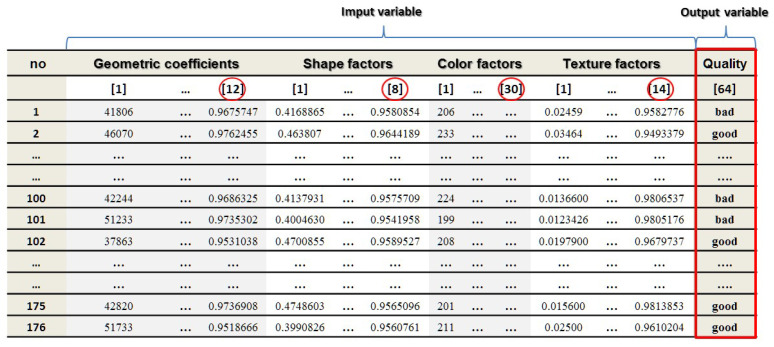
The structure of the training file.

The generated training data file, containing the above-mentioned representative features in its structure, was subsequently used in the process of creating a neural model. The structure of the training data set consisted of 64 input variables describing the geometry, shape factors, color, and texture of barley kernels. The created file contained 176 cases, normally divided in the ratio 2:1:1, into the following subsets: training, validation, and test, respectively. The structure of the training file is shown in Figure 6.

### 2.2. Methods

The relatively large number of descriptors (64) compared to the number of training cases (176 images) may cause difficulties in neural analysis based on discrete optimization methods [19,20]. One of the solutions used in such cases is the use of a statistical method of reducing the input data in the analyzed set, e.g., the standard technique of PCA (Principal Component Analysis).

The methods of discrete compression of digital images are based on the use of the phenomenon of redundancy of information encoded in graphic form and transforming it in such a way as to lead to a new representation of data devoid of mutual stochastic relationships. The consequence of these actions is to obtain a “new” data space with a smaller dimension. Thus, data compression means expressing the initial set of information encoded, for example, in the form of coefficients characterizing the image by means of a representation characterized by a smaller dimension. The technique based on image decomposition in the form of matrix representation is one of the possibilities of frequently used methods (algorithms) of graphic data compression.

The transformation of the proper covariance matrix is performed to remove correlations between adjacent pixels. Practice shows that encoding uncorrelated data produces better results and the information contained in a given coefficient is not duplicated when encoding another representative parameter.

A relatively popular way to reduce the dimension of correlated multivariate data is the standard method of PCA [21]. It determines the directions of the maximum variability of the original input data by rotating the coordinate system in such a way that the maximum variance of the data after the transformation occurs along the new axes. It is expected to retain as much valuable information as possible in the processed data. However, the directions of the maximum variance are not necessarily the directions of the maximum amount of information [20]. In technical sciences (signal processing, artificial neural networks, etc.), this method is referred to as the lossy Karhunen-Loeve transform (lossy KLT). This determines a linear transformation involving the rotation of data to a new coordinate system, formed by the eigenvectors of the covariance matrix, determined for the analyzed data. The eigenvalues (corresponding to individual eigenvectors) determine how the amount of “variation” in the analyzed data is represented by the respective eigenvectors. The innovative approach to the KLT method formalized by the Finnish scientist Erkki Oja is based on the use of a special ANN topology, represented by self-associative neural networks (SANN). The idea of using neural networks implementing *KLT* transformations also allows for the generalization of this method, which is free from the limitation resulting from the linear nature of the transformation matrix. In this way, SANNs are also able to execute a non-linear version of the *KLT* transformation.

Self-associative neural networks (SANNs), usually in the form of linear networks or MLP (multilayer perceptron), have a specific topology. These networks are characterized by an identical number of neurons in both the input and output layers [22,23]. They are therefore intended to reproduce on their outputs the values given at the input. The characteristic feature of the auto-associative network is that the hidden layer contains fewer neurons than the input and output layers. This results in the reduction of the number of data in the input vector.

Networks of this type can be used successfully, mostly to reduce the dimension of the vector representing the input data [24,25], which significantly supports the process of creating an optimal neural topology to solve a given problem. In particular, this technique can be an effective tool for the nonlinear compression of various types of data. In order to perform neural compression (reduction) of empirical data, the module of the *Statistica* v. 10 package was used. The standard procedure was followed with the steps as below:

1. Preparation of a data file for the training of the self-associative network. For this purpose, all the original output variables (i.e., the problem that will be solved after obtaining a compressed representation of the input data) were initially “Omitted” (at the stage of searching for methods of reducing the input data set, the output signals do not matter at all). Then all input variables were treated as output data;
2. Creation of a five-layer *MLP* self-associated network. The middle layer contains significantly fewer neurons than the input or output layer. The other two hidden layers have a relatively large and equal number of neurons;
3. Training of a self-associative neural networks (SANN) on the basis of the training file prepared as described above. For this purpose, the coupled gradients (CG) method was used;
4. Removal of the last two layers in the self-associative neural networks (SANN). After the application of this procedure, the created network represents a structure that converts the primary (numerous) input data into less numerous data in the middle layer (formerly hidden, now output). The “reduced” network executes data processing from the input layer to the output (formerly hidden) layer to perform non-linear dimension reduction;
5. Use of the obtained network to generate a new version of the input data with a reduced dimension. In this way, a representative training file with a reduced dimension of the input data vector is obtained;
6. Finally, the creation of a new network solving the fundamental problem and then conducting the process of training it, using a data file with a reduced dimension.

**Figure 7:**
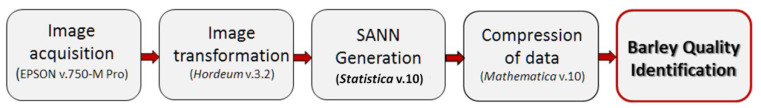
Schematic procedure of neural classification.

The schematic procedure of neural classification is presented in Figure 7.

For designing the neural models, an artificial neural network simulator, implemented in the statistical package *Statistica* v.10 suite, was used. Creating of the neural models was conducted in two stages. In the first stage, the efficient “Automatic network designer” implemented in the statistical environment was used. This tool allowed for the automation and simplification of initial network set searching procedures that would best model the analyzed process. During the second stage, the “User network designer” tool was used. This tool was used repeatedly, modifying initial parameter-related settings and learning algorithms and the network structure itself [26].

## 3. Results and Discussion

**Figure 8:**
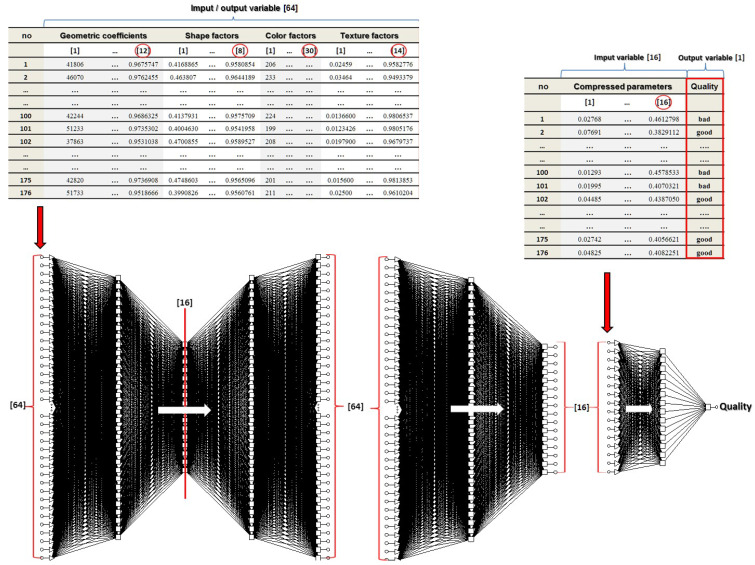
Structure of the simulation procedure.

The created MLP (multilayer perceptron) neural model was trained with the use of optimization algorithms implemented in the *Statistica* package v.10 [5]. According to the procedure implemented to the “Neural Networks” module, the learning process of the auto-associative network was conducted using the standard CG (conjugate gradients) algorithm. The optimization of SANN with the structure: 64:32:16:32:64 was realized in 1000 epochs. Then, the generated network was divided according to its symmetry axis (three hidden layers, Figure 8). The reduced, three-layer MLP SNN: 64-32-16 was further used to generate a new data set consisting of 16 compressed input variables and 176 training cases [27].

Subsequently, the second MLP network (solving the main problem) was created and trained. A data set with a reduced dimension (16 descriptors) was used. For this purpose, the “Automatic designer” IT tool implemented in the Statistica v. 10 package was used. The training of the reduced neural model was performed using the BP (back propagation) algorithm, realized in 1000 epochs. The MLP topology 16-14-1 turned out to be the best neural network.

One-way MLP neural networks are among the best researched network topologies that are most commonly used in practice. The MLP multilayer perceptron represents a class of so-called parametric neural models [28]. It is a one-way, multi-layer neural network taught by means of the “with teacher” technique. It is characterized by the number of neurons constituting its structure being significantly smaller than the number of cases of the training file.

The most frequently used measure of the performance of an artificial neural network is the total error known as the *RMS* (*Root Mean Square*) error created by the generated model on the training file (training, testing, and validation) [29]. It is determined by summing the squares of individual errors, dividing the obtained sum by the number of used cases, and then determining the square root from the obtained quotient as below:

(1) $$RMS=\sqrt{\frac{\sum_{i=1}^{n}{\left(y_{i}-z_{i}\right)}^{2}}{n}}$$

*n*—number of cases,

*y_i_*—real values,

*z_i_*—values determined using the network.

The *RMS* error is usually the most interpretable single value for the summary network error. The quality of the neural network generated as above should be considered good as *RMS* error for MLP: 16-14-1 was respectively:

- 0.051272 for the training file;
- 0.064537 for the validation file;
- 0.073453 for the test file.

The similar values and low *RMS* error for the training, validation, and test file prove good generalization properties of the resultant ANN. Its small value, in turn, implies good classification properties of the generated model. The model structure of the generated synergistic MLP classifier and the scheme of the simulation process are shown in Figure 8.

The created hybrid neural model was taught with the use of optimization algorithms implemented in the Statistica v.10 package [11,12].

Table 3 presents a set of top 10 generated neural classifiers.

## 4. Conclusions

Neural modeling and image analysis methods for identifying the quality of malting barley were found to be effective tools used in supporting the decision-making processes occurring during beer production. Quality identification of malting barley based on digital photos of malting barley kernels was best performed by a reduced neural network of the multilayer (MLP: 16-14-1) perceptron type. The conducted analysis allowed us to state that, for the correct qualitative classification of malting barley, it is enough to know 64 representative parameters describing 176 images of kernels. The conducted research allowed for the formulation of the following conclusions:

1. The obtained research results confirm the hypothesis that artificial neural networks and image analysis techniques are effective tools supporting the process of quick and reliable qualitative identification of malting barley kernels based on their digital images;
2. A neural reduction of the dimension of the variable vector was found necessary due to the small number of obtained photos of kernels (176) compared to the number of descriptors (64);
3. Qualitative analysis of the reduced neural model showed the best classification ability for the topology MLP: 16-14-1 taught on the basis of a compressed training file (with the number of descriptors reduced to 16);
4. The conducted research indicates the usefulness of the developed model as an instrument that effectively supports the decision-making processes occurring during beer production.

## Figures and Tables

**Table 1:** Main technological features of selected varieties of malting barley [7]. The bold is the leading result.

	Specification	Synthetic Evaluation of Brewing Value	Extractivity	Wort Viscosity	Kolbach Index
Variety		Scale: 9°			
*Beatrix*		5.10	3	7	7
***Sebastian***		**6.85**	**7**	**7**	**6**
*Xanadu*		6.70	7	7	6
*Prestige*		5.90	5	7	6
*Tiffany*		3.80	2	5	5
*Wintmalt*		4.10	2	6	6

**Table 2:** Types of pollutants included in the “Hordeum 3.2” IT system. The blackgroud is the copyright shaders.

Type of Pollution
1. Decorticated
2. Halves
3. Grain with the embryo stamped
4. Damp
5. Overgrown
6. Grain affected by pests
7. Grain sprouted
8. The grain is affected by mold
9. Inorganic material
10. Organic material
11. Toxic, harmful seeds
12. Other grains/seeds

**Table 3:** A set of the best 10 generated neural classifiers.

Type SSN	Inputs	Hidden Neurons	Training Error	Validation Error	Test Error	Training Algorithm
Linear	16	-	0.07748300	0.07492575	0.08026012	PI
RBF	16	7	0.07882754	0.08332683	0.08940989	KM.KN.PI
RBF	16	8	0.07000708	0.07293875	0.07921950	KM.KN.PI
RBF	16	16	0.07869445	0.08290467	0.08045218	KM.KN.PI
RBF	16	10	0.06399839	0.07243709	0.07048332	KM.KN.PI
MLP	16	1	0.06215839	0.06040448	0.07438998	BP50.CG54
MLP	16	34	0.06328137	0.06965351	0.06457506	BP8
MLP	16	52	0.06377303	0.07956288	0.07524478	BP2
MLP	16	79	0.05684823	0.05950489	0.05833947	BP0
ML P	16	14	0.05127200	0.06453700	0.07345300	BP23

RBF—radial basis function; MLP—multilayer perceptron; PI—pseudo-inversion, linear least-squares optimization; KMK—means, the assignment of centers; KN—K-nearest neighbor, the assignment of deviations; BP50—back propagation—50 training epochs; CG54—conjugate gradient descent—54 training epochs.

## References

[1] Przybył K., Pilarska A., Duda A., Wojcieszak D., Frankowski J., Koszela K., Boniecki P., Kujawa S., Mueller W., Zaborowicz M. Health properties and evaluation of quality of dried strawberry fruit produced using the convective drying method with neural image analysis. Proceedings of the Eleventh International Conference on Digital Image Processing (ICDIP 2019).

[2] Mildner-Szkudlarz S., Bajerska J., Górnaś P., Segliņa D., Pilarska A., Jesionowski T. (2016). Physical and bioactive properties of 305 muffins enriched with raspberry and cranberry pomace powder: A promising application of fruit by-products rich in bio- 306 compounds. Plant Foods Hum. Nutr..

[3] Janczak D., Lewicki A., Mazur R., Boniecki P., Dach J., Przybyl J., Pawlak M., Pilarski K., Czekala W. The Selected Examples of the Application of Computer Image Analysis in the Assessment of Environmental Quality. Proceedings of the Fifth International Conference on Digital Image Processing (ICDIP 2013).

[4] Boniecki P., Zaborowicz M., Pilarska A., Piekarska-Boniecka H. (2020). Identification process of selected graphic features apple 298 tree pests by neural models type MLP, RBF and DNN. Agriculture.

[5] Boniecki P., Piekarska-Boniecka H., Koszela K., Nowakowski K., Kujawa S., Majewski A., Weres J., Raba B. (2015). Neural 300 identification of selected apple pests. Comput. Electron. Agr..

[6] Zaborowicz M., Boniecki P., Koszela K., Przybyl J., Mazur R., Kujawa S., Pilarski K. Use of Artificial Neural Networks in the Identification and Classification of Tomatoes. Proceedings of the Fifth International Conference on Digital Image Processing (ICDIP 2013).

[7] Nowakowski K., Boniecki P., Tomczak R.Ł., Raba B. Identification process of corn and barley kernels damages using neural image analysis. Proceedings of the 3rd International Conference on Digital Image Processing (ICDIP 2011).

[8] Pilarska A.A., Boniecki P., Idzior-Haufa M., Zaborowicz M., Pilarski K., Przybylak A., Piekarska B. (2021). Image analysis methods in classifying chosen malting barley varieties. Agriculture.

[9] Szczypiński P.M., Zapotoczny P. (2012). Computer vision algorithm for barley kernel identification, orientation estimation and surface structure assessment. Comput. Electron. Agric..

[10] Kociołek M., Szczypiński P.M., Klepaczko A. Preprocessing of barley grain images for defect identification. Proceedings of the Signal Processing: Algorithms, Architectures, Arrangements, and Applications (SPA).

[11] Ramirez-Paredes J.-P., Hernandez-Belmonte U.-H. (2020). Visual quality assessment of malting barley using color, shape and texture descriptors. Comput. Electron. Agric..

[12] Kozłowski M., Gorecki P., Szczypinski P.M. (2019). Varietal classification of barley by convolutional neural networks. Biosys. Eng..

[13] Stejskal V., Vendl T., Li Z., Aulicky R. (2020). Efficacy of visualevaluation of insect-damagedkernels of maltingbarley by *Sitophilusgranarius* from variousobservationperspectives. J. Stored Prod. Res..

[14] Lopes J.F., Ludwig L., Barbin D.F., Grossmann M.V.E., Barbon S. (2019). Computer vision classification of barley flour based on spatial pyramid partition ensemble. Sensors.

[15] Mundt M., Koeppe A., Bamer F., David S., Markert B. (2020). Artificial Neural Networks in motion analysis—Applications of unsupervised and heuristic feature selection techniques. Sensors.

[16] Kim J., Lee C., Park S. (2017). Artificial Neural Network-Based Early-Age concrete strength monitoring using dynamic response signals. Sensors.

[17] Raba B. (2014). Determination of Malting Barley Grain Impurities Using Computer Image Analysis and Artificial Intelligence Methods. Ph.D. Thesis.

[18] Karlović A., Jurić A., Ćorić N., Habschied K., Krstanović V., Mastanjević K. (2020). By-products in the malting and brewing industries—re-usage possibilities. Fermentation.

[19] Boniecki P., Koszela K., Piekarska-Boniecka H., Nowakowski K., Przybyl J., Zaborowicz M., Raba B., Dach J. Identification of selected apple pests, based on selected graphical parameters. Proceedings of the 5th International Conference on Digital Image Processing (ICDIP 2013).

[20] Boniecki P., Nowakowski K., Tomczak R.Ł. Neural networks type MLP in the process of identification chosen varieties of maize. Proceedings of the 3rd International Conference on Digital Image Processing (ICDIP 2011).

[21] Qiu J., Wang H., Lu J., Zhang B., Du K.L. (2012). Neural Network Implementations for PCA and Its Extensions. ISRN Artif. Intell..

[22] Lee D., Choi M., Lee J. (2021). Prediction of Head Movement in 360-Degree Videos Using Attention Model. Sensors.

[23] Geng C., Sun Q., Nakatake S. (2020). Implementation of Analog Perceptron as an Essential Element of Configurable Neural Networks. Sensors.

[24] Fausett L. (1994). Fundamentals of Neural Networks.

[25] Bishop C. (1995). Neural Networks for Pattern Recognition.

[26] Boniecki P., Dach J., Nowakowski K., Jakubek A. Neural image analysis of maturity stage during composting of sewage sludge. Proceedings of the International Conference on Digital Image Processing (ICDIP 2009).

[27] Nowakowski K., Boniecki P., Dach J. The identification of mechanical damages of kernels basis on neural image analysis. Proceedings of the International Conference on Digital Image Processing (ICDIP 2009).

[28] Schmidhuber J. (2014). Deep Learning in Neural Networks: An Overview.

[29] Przybylak A., Boniecki P., Koszela K., Ludwiczak A., Zaborowicz M., Lisiak D., Stanisz M., Slosarz P. (2016). Estimation of intramuscular level of marbling among Whiteheaded Mutton Sheep lambs. J. Food Eng..

